# Bolstering Skin Grafts With a Surgical Scrub Brush: A Cost-effective Solution

**Published:** 2017-07-07

**Authors:** Mitchell Buller, Thomas J. Lee, Jared Davis, Bradon J. Wilhelmi

**Affiliations:** Division of Plastic and Reconstructive Surgery, University of Louisville, Louisville, Ky

**Keywords:** skin graft, scrub brush, bolster, stent, foam

## Abstract

**Objective:** The objective of this article is to review the methods currently used for the bolstering of skin grafts and compare their advantages and disadvantages with those of the dry, sterile surgical scrub brush. We report a series of cases performed at a single institution and compare the cost-effectiveness, application, and limitations of this method with other options for skin graft bolstering. **Methods:** A PubMed search using the parameters “(bolster) AND skin graft” was conducted, yielding 85 results. A total of 40 publications met the criteria for our literature review. The costs of the foam bolsters utilized as stents for skin grafts were obtained from the Central Supply and Resource Division of the University of Louisville Hospital for a cost analysis. The cost per square centimeter of each bolster material was calculated. **Results:** At $0.003/cm^2^, the 3M Reston foam is the most inexpensive of the 3 bolster materials analyzed. The dry, sterile surgical scrub brush has a similar cost at $0.006/cm^2^ but carries the advantage of sterility. The material cost of negative pressure wound therapy is $0.47/cm^2^, and the cost of the system as a whole makes it a much more expensive alternative. In 6 patients with defects of varying size and location, the scrub brush bolster showed a near 100% graft take and no complications. **Conclusions:** The dry, sterile surgical scrub brush presents a readily available and low-cost option for the stenting of small skin grafts and should be considered a viable method in the armamentarium of available skin graft bolsters.

**Objective:** Skin grafting is a commonly used and dependable method on the reconstructive ladder. Success in skin grafting requires a suitable donor site and postoperative prevention of factors that can cause graft failure, namely, shear, fluid accumulation, and infection. The ideal dressings minimize graft movement, apply pressure to prevent minimal fluid from building underneath the graft, and keep the site clean to minimize the amount of bacterial exposure. The methods of graft dressings have evolved over the years, and a wide range of supplies are available for that purpose. Traditional methods utilize a cotton or gauze bolster secured by silk ties, whereas new innovations such as the wound VAC (KCI, San Antonio, Tex) allow for negative pressure therapy. A commonly used material for graft immobilization at our institution is Reston self-adhering foam (3M Company, St Paul, Minn).

This method was first discussed by Weiner and Moberg,^1^ who described a multilayered stent consisting of adaptive nonadherent gauze, moist cotton gauze, and Reston foam affixed with stainless steel staples. The mechanical properties of the synthetic urethane foam allow for uniform pressure distribution over the graft bed, whereas its porous surface provides for absorption of exudate. In vitro testing demonstrated that the foam exerted a pressure between that of intracapillary and interstitial pressure, permitting adequate tissue perfusion and preventing accumulation of excess fluid.

They conclude that this method creates the ideal stent, which has specific characteristics. These characteristics are (1) consistent reliability in achieving 100% graft take, (2) case and expediency of surgical application, (3) technical control of the pressure exerted by the stent, (4) uniform distribution of the pressure over the entire graft bed, (5) intrinsic elastic properties that exert pressure in a desired physiologic range, and (6) immediate accessibility to stent material.^1^

Of particular concern to us is the requirement of immediate accessibility to stent material. While Reston foam has many advantages, it also requires sterilization prior to use. However, the manufacturer does not recommend sterilizing Reston foam and discourages its use as a postoperative compression dressing.^2^

The use of sponge from a dry, sterile surgical scrub brush has been previously described and presents an inexpensive and readily available alternative to other foam products.^3^ Its physical properties are similar to other foams used in previous studies.^1^^,^^4^^-^6 We report a series of cases performed at the University of Louisville Hospital and compare the cost-effectiveness, application, and limitations of this method with other options for skin graft bolstering.

## METHODS

### Literature review

A PubMed search using the parameters “((bolster[Title]) AND graft[Title]) AND skin” was performed, yielding only 7 results. The search was then expanded using the parameters “(bolster) AND skin graft,” which yielded 85 results. A filter of “human’ subjects only” was applied, leaving 71 results. Results were reviewed and our discussion is limited to immobilization methods used for the broad application of common skin grafts. Publications referencing bolsters created only for use in specific graft sites or discussing the impregnation of bolsters with antibiotics or other fluids were excluded.

These exclusions left a total of 40 publications.

### Cost analysis

The cost of 3 foam bolsters utilized for skin grafts was obtained from the Central Supply and Resource Division of the University of Louisville Hospital for a cost analysis. These products are the BD CareFusion dry, sterile surgical scrub brush,^7^ the KCI V.A.C. GranuFoam Small Dressing Kit,^8^ and 3M Reston Self-Adhering Foam.^9^ The hospital cost per square centimeter of each bolster material was calculated and is shown in Table 1.

### Case series

A series of 8 cases using the dry, sterile surgical scrub brush as a bolster for skin grafts was performed at the University of Louisville Hospital.

## RESULTS

### Case example

**Figure 1:**
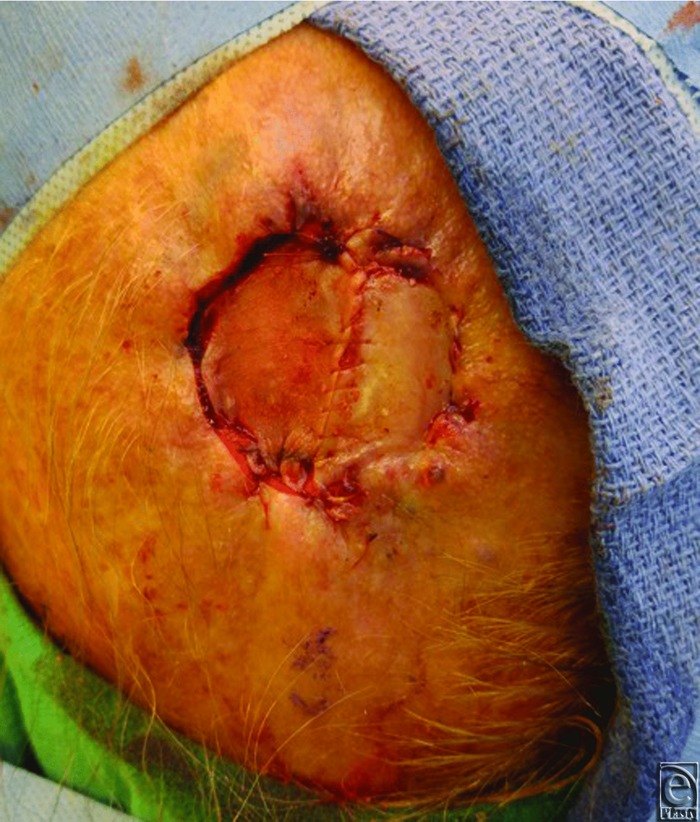
Coverage of melanoma excision defect with split-thickness skin graft.

**Figure 2:**
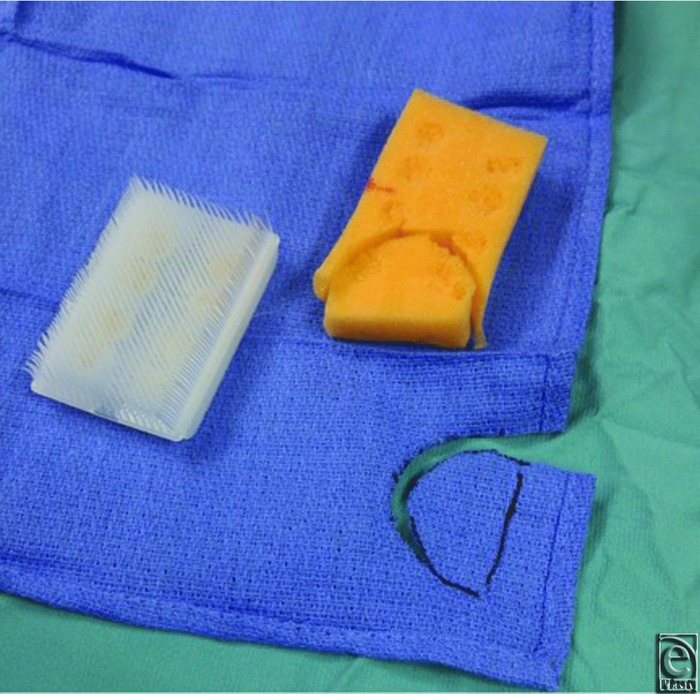
Modification of dry, sterile scrub brush for use as a bolster for the skin graft.

**Figure 3:**
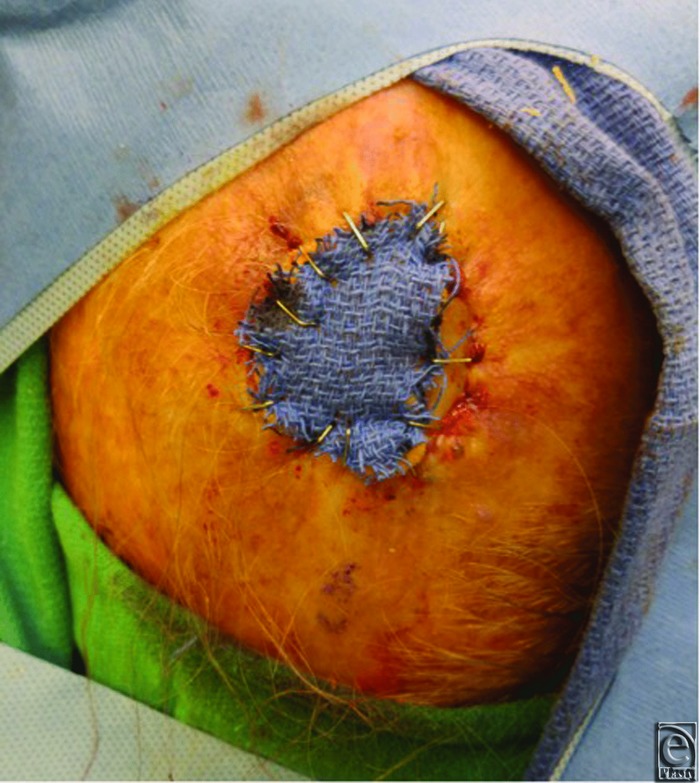
Attachment of the dry, sterile scrub brush bolster to the skin graft using surgical staples.

D.G. was an 84-year-old man with a growing lesion on the right temporal scalp that was found to be a melanoma by punch biopsy. He was taken to the operating room for wide local excision, which left an initial defect measuring 5 × 5 cm. An adjacent tissue rearrangement was performed that minimized the defect to 2 × 2 cm, which was then covered with a split-thickness skin graft. He had no complications from the procedure, and his skin graft healed well. Final pathology, however, showed a focus of melanoma at one of the margins, so he was taken back to the operating room for reexcision. This left a defect measuring 4 × 3 cm, which was subsequently covered using a split-thickness skin graft seen in Figure 1. A sterile scrub brush and a blue towel were used to create the bolster and secured to the surrounding skin with staples, as seen in Figures 2 and 3. He was sent home with the bolster and returned to clinic 5 days postoperatively for removal. He had complete graft take without any complications. The other patients in this series had similar outcomes, with maximal defect length of less than 5 cm. The full list of patients in the case series can be found in Table 2.

## DISCUSSION

The tie-over-bolster technique is one of the oldest methods for bolstering skin grafts. Individual sutures with long tails are placed along the periphery of the graft site, and a bolster is placed on the graft. This bolster is typically made using a nonadherent dressing layered on top with a soft, pliable, and absorbent material such as cotton balls or sterile gauze. The sutures are then tied over the top of the bolster to secure it in place. This method can be considerably time-consuming, and the tightness of the sutures over the bolster can create uneven pressure across the graft bed, as well as excess tension on the edges where the sutures are placed. To address these issues, modifications of the tie-over-bolster method were developed, including various suture methods such as running sutures or those placed in specific patterns.^10^^-^13 Other modifications such as a device to manipulate pressure and the use of a barbed suture have also been proposed.^14^^,^^15^ These adaptations make it faster and more efficient while providing a more consistent pressure across the graft bed. Staples are an effective and quick method to secure the bolster; however, they are significantly more costly than suture. In addition, if left on the skin for too long, they can leave unsightly scarring and local erythema.

The use of foam for skin graft immobilization has been previously described.^3^ The use of self-adhering foam has been documented on multiple occasions.^1^^,^^5^^,^^6^^,^^16^ Others have used simple polyurethane sponge as a bolster, which has been shown to be more effective than the traditional tie-over-bolster method.^4^

Vacuum-assisted closure (VAC), a relatively newer technology, is particularly effective for use on large grafts, areas that are more susceptible to fluid accumulation such as the perineum and areas that are difficult to bolster with other methods such as large hand or foot burns. This technique uses a polyurethane sponge and applies a negative pressure, helping suction away any fluid that has accumulated. Studies have found that this technique allows for an excellent graft take with decreased local edema and has compared favorably with standard bolster dressings.^17^^-^20

A variety of other materials have been also used to cover new skin grafts. A unique method of internal bolstering has been characterized for wounds where the skin graft lies in a cavity.^21^ Novel materials such as silicone^22^ and thermoplastic materials^23^ have been used successfully. Simple dressings^24^^-^27 have shown promise when compared with pressure dressings and have demonstrated fewer residual marks upon healing of the graft. Mesh gauze^28^ and cotton balls^29^ have also proven useful. This multitude of methodologies illustrates the uniqueness of each patient with skin graft and the creativity required of each surgeon.

Reston foam is an inexpensive bolster sold in nonsterile packaging that is not safe to be sterilized in an autoclave. This creates a potential risk for contamination and subsequent infection. They also come only in one size, which can become problematic in terms of storage in facilities with limited space such as a surgery center. In addition, Reston foam has limited utility other than for bolstering, whereas the surgical scrub bush can be used for its primary purpose in the operating room environment.

Negative pressure therapy is certainly the most expensive method. The sponge itself is more expensive than the 2 alternatives, and it also requires the use of the proprietary impermeable adhesive dressing and the device to create the vacuum. This adds significant cost on a daily basis, since at our institution there is a daily rental fee. Portable machines exist, which allows their use in both inpatient and outpatient settings.

The use of foam from the surgical scrub brush is not without its limitations, the most obvious being size. Dimensions are approximately 82-mm long, 48-mm wide, and 19-mm thick. This makes it ideal for use on smaller grafts; however, for wounds larger than the area of the sponge, multiple sponges can be used, but the uniformity of the bolster pressure may be at risk.

## CONCLUSIONS

The dry, sterile surgical scrub brush presents a readily available and low-cost option for the stenting of small skin grafts and should be considered a viable method in the armamentarium of available skin graft bolsters.

## Figures and Tables

**Table 1:** Cost analysis of common foams used for skin graft immobilization

	Width	Length	Thickness	Cost	Cost/cm^2^
3M Reston foam	7.875	11.750	0.438	$1.80	$0.003
BD scrub brush	1.875	3.250	0.750	$0.22	$0.006
KCI V.A.C. kit	2.953	3.937	1.260	$35.13	$0.468

**Table 2:** Summary of patients in case series

Age	Gender	Location	Size, cm	Graft take	Complications
84	M	Scalp	2 × 2	100%	None
76	M	Scalp	4 × 4	100%	None
80	M	Scalp	3 × 5	100%	None
72	F	Left small finger	2 × 2	100%	None
45	M	Left thumb	3 × 1	100%	None
68	F	Scalp	4 × 5	100%	None
44	F	Right ear	5 × 2	100%	None
65	M	Scalp	3.5 × 4.5	100%	None

## References

[1] Weiner LJ, Moberg AW (1984). An ideal stent for reliable and efficient skin-graft application. Ann Plast Surg.

[2] *3M Reston Self-Adhering Foam Products: Commonly Asked Questions* (2005).

[3] Egan CA, Gerwels JW (1998). Surgical pearl: use of a sponge bolster instead of a tie-over bolster as a less invasive method of securing full-thickness skin grafts. J Am Acad Dermatol.

[4] De Gado F, Chiummariello S, Monarca C (2008). Skin grafting: comparative evaluation of two dressing techniques in selected body areas. In Vivo.

[5] Harris D (1981). A new technique of skin-grafting using Steri-Greffe and a self-adhering foam pad. Br J Plast Surg.

[6] Wells MD, Kirn DS (1995). A new method of skin-graft stabilization—the Reston technique. Ann Plast Surg.

[7] *Focus on Quality Care Product Resource Guide*

[8] V.A.C. GranuFoam Standard Dressing Kits (2013). http://www.kci-medical.ie/IE-ENG/vacgranufoamstandarddressingskits.

[9] *3M Reston Self-Adhering Foam Products* (2009).

[10] Adams DC, Ramsey ML, Marks VJ (2004). The running bolster suture for full-thickness skin grafts. Dermatol Surg.

[11] Branfman GS, Cassel JM (1988). A simple method for securing a bolster in position over a split-thickness skin-graft. Plast Reconstr Surg.

[12] Gandhi V, Khurana S, Aggarwal P (2005). Surgical pearl: use of a new suturing technique to bolster partial and full thickness skin grafts. J Am Acad Dermatol.

[13] Srivastava D, Kouba DJ (2009). A “Lilliputian” technique for rapid and efficient securing of bolster dressings over full-thickness skin grafts. Dermatol Surg.

[14] Amir A, Sagi A, Fliss DM, Rosenberg L (1996). A simple, rapid, reproducible tie-over dressing. Plast Reconstr Surg.

[15] Joyce CW, Joyce KM, Mahon N (2014). A novel barbed suture tie-over dressing for skin grafts: a comparison with traditional techniques. J Plast Reconstr Aesthet Surg.

[16] Larson PO (1990). Foam-rubber stents for skin-grafts. J Dermatol Surg Oncol.

[17] Hanasono MM, Skoracki RJ (2007). Securing skin grafts to microvascular free flaps using the vacuum-assisted closure (VAC) device. Ann Plast Surg.

[18] Isago T, Nozaki M, Kikuchi Y, Honda T, Nakazawa H (2003). Skin graft fixation with negative-pressure dressings. J Dermatol.

[19] Moisidis E, Heath T, Boorer C, Ho K, Deva AK (2004). A prospective, blinded, randomized, controlled clinical trial of topical negative pressure use in skin grafting. Plast Reconstr Surg.

[20] Scherer LA, Shiver S, Chang M, Meredith JW, Owings JT (2002). The vacuum assisted closure device—a method of securing skin grafts and improving graft survival. Arch Surg.

[21] Mardini S, Chen HC, Salgado CJ, Chung TT, Ozkan O, Lin SL (2006). The balloon as an internal bolster for skin grafts. Ann Plast Surg.

[22] Roh MR, Shin JU, Chung KY (2008). Silicone net bolster dressing for skin grafts. Dermatol Surg.

[23] Meads SB, Greenway HT, Eaton JS (2006). Surgical pearl: thermoplastic bolster dressing for full-thickness skin grafts. J Am Acad Dermatol.

[24] Dhillon M, Carter CP, Morrison J, Hislop WS, Currie WJR (2015). A comparison of skin graft success in the head & neck with and without the use of a pressure dressing. J Maxillofac Oral Surg.

[25] Langtry JAA, Kirkham P, Martin IC, Fordyce A (1998). Tie-over bolster dressings may not be necessary to secure small full thickness skin grafts. Dermatol Surg.

[26] Pulvermacker B, Chaouat M, Seroussi D, Mimoun M (2008). Tie-over dressings in full-thickness skin grafts. Dermatol Surg.

[27] Shimizu I, MacFarlane DF (2013). Full-thickness skin grafts may not need tie-over bolster dressings. Dermatol Surg.

[28] Park S (1998). Mesh-over dressing: Tie-over dressing without tie-over. Plast Reconstr Surg.

[29] Schade VL, Roukis TS (2010). Sterile cast padding as an alternative to commercially available cotton balls for split-thickness skin graft bolster dressing. J Foot Ankle Surg.

